# The partial replacement of sodium chloride with sodium bicarbonate or sodium sulfate in laying hen diets improved laying performance, and eggshell quality and ultrastructure

**DOI:** 10.1016/j.psj.2021.101102

**Published:** 2021-03-12

**Authors:** Yu Fu, Jing Wang, Hai-jun Zhang, Shu-geng Wu, Jian-min Zhou, Guang-hai Qi

**Affiliations:** Key Laboratory of Feed Biotechnology of Ministry of Agriculture & Rural Affairs, Feed Research Institute, Chinese Academy of Agricultural Sciences, Beijing 100081, China

**Keywords:** Chloride, eggshell quality, laying hen, laying performance, ultrastructure

## Abstract

This study investigated the effects of dietary chloride (**Cl**) reduction on laying performance and eggshell quality by substitution of sodium bicarbonate (NaHCO_3_) or sodium sulfate (Na_2_SO_4_) for part of dietary sodium chloride (NaCl), and further explored its mechanism for improving eggshell quality. A total of 360 29-wk-old Hy-line Brown laying hens were randomly allocated to 5 dietary treatments, including a basal diet contained 0.33% NaCl (control group, 0.27% dietary Cl), and 4 experimental diets that contained 0.21% and 0.15% dietary Cl by substituting Na_2_SO_4_ or NaHCO_3_ for part of NaCl in the basal diet. No significant differences were observed in blood Na^+^, Cl^−^, K^+^ and Ca^2+^ levels and pH value as well as serum creatinine and uric acid contents among 5 treatments (*P* > 0.05). Dietary Cl reduction increased egg production and ADFI during wk 33 to 36, 37 to 40 and 29 to 40 of age and decreased feed conversion ratio during wk 37 to 40 of age (*P* < 0.05). The hens fed with diets containing 0.15% Cl increased eggshell breaking strength, thickness and weight ratio in wk 40 of age (*P* < 0.05). Birds fed with dietary 0.21% and 0.15% Cl exhibited higher effective layer thickness and lower mammillary layer thickness of eggshell than those fed with dietary 0.27% Cl (*P* < 0.05). Apparent Ca metabolizability of hens was increased with dietary Cl reduction (*P* < 0.05). Total Ca of eggshell of dietary 0.15% Cl group was higher than that of dietary 0.27% Cl group (*P* < 0.05). No significant differences in laying performance, eggshell quality and Ca metabolism of layers were observed between Na_2_SO_4_ and NaHCO_3_ replacement groups (*P* > 0.05). Overall, dietary Cl reductions could improve laying performance and eggshell quality by substitution of NaHCO_3_ or Na_2_SO_4_ for part of NaCl, and there were no differences in the improvements between these two substitutes. The improved eggshell quality may be attributed to improved eggshell ultrastructure and increased supply of eggshell Ca_2_CO_3_.

## INTRODUCTION

Chloride (**Cl**) is an essential macro-mineral element for poultry. The concentrations, chemical properties, currents and osmotic pressures of Cl^−^ and other non-metabolizable inorganic ions, such as sodium (**Na**), potassium (**K**) and calcium (**Ca**) ions, jointly contribute to maintain the pH value and ions homeostasis of blood (Mushtaq and Pasha, 2013). Thus, it is critical to supply them in precise levels and adequate balance. The recommended level of dietary Cl for laying hens is 0.15% (NRC, 1994), generally provided by approximately 0.33% sodium chloride (**NaCl**) in practice. In addition to NaCl, the use of hydrochloride additives such as lysine hydrochloride or choline chloride may result in high Cl levels in practical diets (0.25%, Wang et al., 2020; 0.27%, Fu et al., 2021).

Eggshell quality is a vital economic trait as about 7% eggs were lost before their intended use due to shell damage (Hamilton et al. 1979). During eggshell formation, Ca^2+^ and HCO_3_^−^ ions are continuously supplied to the eggshell gland from the blood capillary via transepithelial transport (Jonchère et al., 2012). This process highlights the importance of carbonic anhydrases which can catalyze the hydration of carbon dioxide to HCO_3_^−^ in an alkaline condition (Supuran, 2008). However, higher levels of dietary Cl may impair the synthesis of HCO_3_^−^ by inhibiting the carbonic anhydrase activity due to their indirect acidogenic effect (Murakami et al., 2001). Besides, in terms of eggshell quality, there was an obvious interaction between Ca and Cl, and dietary Ca supplementation could alleviate the reduction of eggshell thickness in the high Cl level group (dietary 0.86% Cl level) (Austic and Keshavarz, 1988). This indicates that Ca^2+^ supply may also be restricted during the eggshell formation in the group receiving high dietary Cl. Therefore, it is quite necessary to explore more suitable methods of salt addition to avoid potentially harmful effects of high Cl on eggshell quality.

A Cl-free salt may be more suitable for diets to improve eggshell quality (Faria et al., 2000). Sodium bicarbonate (**NaHCO_3_**) and sodium sulfate (**Na_2_SO_4_**) are common Na salts for laying hens, and the acidity of HCO_3_^−^ and SO_4_^2−^ is weaker than Cl^−^ (Halley et al., 1987). It has been reported that dietary NaHCO_3_ or Na_2_SO_4_ addition improved laying performance (Yörük et al., 2004; Wei et al., 2015) and eggshell quality (Balnave and Muheereza, 1997; Ghorbani and Fayazi, 2009; Elsheikh and Salama, 2010; Wei et al., 2015). Therefore, the replacement of NaCl with NaHCO_3_ or Na_2_SO_4_ would be the more suitable method of salt supplement. However, our previous study found that it could impair laying performance and eggshell quality for laying hens when NaCl was completely replaced by Na_2_SO_4_, and the recommended dietary Cl level should not be less than 0.15% (Wang et al., 2020).

The objective of this study was to compare the effects of dietary Cl reduction on laying performance and eggshell quality by substitution of NaHCO_3_ or Na_2_SO_4_ for part of NaCl, and further explore its possible mechanism. This study would provide references for the salt addition in laying hen diets.

## MATERIALS AND METHODS

### Experimental Design and Diets

This study was conducted under the management of the Animal Care and Use Committee of the Feed Research Institute of the Chinese Academy of Agricultural Sciences. 360 28-wk-old Hy-line Brown laying hens were fed a basal diet (Table 1) which contained 0.33% NaCl (the commercial level; 0.27% dietary Cl) for 1 wk and then randomly allocated to 5 dietary treatments for 12 wk. The control group was sequentially fed the basal diet which contained 0.27% dietary Cl. The other 4 treatment groups were fed the diets contained 0.21% and 0.15% dietary Cl by replacing part of NaCl in the basal diet with Na_2_SO_4_ (≥ 32.4%, [wt/wt] Na, Nafine Chemical Industry Group Co., Ltd., Yuncheng, Shanxi, China) or NaHCO_3_ (≥ 27.1%, [wt/wt] Na, Xilinguole Sonid Alkali Industry Co., Ltd., Xilinguole, Inner Mongolia, China) (Table 2). The Na level of the basal diet was set to be 0.15% according to the nutrient requirements of the National Research Council (1994) and Chinese Feeding Standard of Chicken (Ministry of Agriculture of China, 2004). Each treatment had 6 replicates, with 12 hens in 4 adjacent cages per replicate, totaling 72 hens. All hens were fed in a fully enclosed house that the temperature was controlled at 15 ∼ 23°C, and the humidity was controlled at 60% ∼ 65%. All light sources were kept the light density at 20 lux and applied for 16 h daily. The drinking water contained 64.7 mg/L Na, 2.81 mg/L Cl, and 0.592 mg/L K.

**Table 1:** Dietary composition and nutrient level of the basal diet for laying hens

Ingredient	%	Nutrient level	%
Corn	58.71	AME (MJ/kg)	11.24
Soybean meal	25.60	Crude protein	16.50
Soybean oil	2.00	Calcium	3.52
Limestone	9.00	Methionine	0.36
DL-Methionine	0.11	Lysine	0.83
50% choline chloride	0.12	Total phosphorus	0.53
Calcium hydrogen phosphate	1.30	Available phosphorus	0.33
Yeast culture using distiller's grains	1.50	Methionine +Cysteine	0.65
Montmorillonite	0.10		
Vitamin and mineral premix^1^	0.23		
Sodium Chloride	0.33		
Zeolite powder	1.00		
Total	100.00		

1 Provided per kilogram of diet: vitamin A 12 500 IU; vitamin D_3_ 4 125 IU; vitamin E 15 IU; vitamin K 2 mg; thiamine 1 mg; riboflavin 8.5 mg; calcium pantothenate 50 mg; niacin 32.5 mg; pyridoxine 8 mg; biotin 2 mg; folic acid 5 mg; vitamin B_12_ 5 mg; choline 500 mg; Zn 66 mg; Mn 65 mg; I 1 mg; Fe 60 mg; Cu 8 mg; Se 0.3 mg.

**Table 2:** Experimental design and the contents of sodium (Na) and chloride (Cl) of laying hens’ diets (%)^1^

	Dietary supplementation level				
Treatments	NaCl	NaHCO_3_	Na_2_SO_4_	Dietary Cl content^2^	Dietary Na content^2^
Control (Dietary 0.27% Cl)	0.33			0.27(0.26)	0.15(0.152)
NaHCO_3_ replacement groups					
Dietary 0.21% Cl	0.23	0.14		0.21(0.21)	0.15(0.152)
Dietary 0.15% Cl	0.13	0.29		0.15(0.14)	0.15(0.152)
Na_2_SO_4_ replacement groups					
Dietary 0.21% Cl	0.23		0.12	0.21(0.21)	0.15(0.152)
Dietary 0.15% Cl	0.13		0.24	0.15(0.14)	0.15(0.152)

1 Zeolite powder was used to adjust the total ratio of the diet.

2 The contents of dietary Cl and Na were calculated according to Tables of Feed Composition and Nutritive Value in China (2017, 28th edition) and analyzed by China National Standard Recommendation Assays (Cl: GB/T 6439-2007, State Administration for Quality Supervision and Inspection and Quarantine, 2007; Na: GB/T 13885-2003, State Administration for Quality Supervision and Inspection and Quarantine, 2003). Numbers in parentheses are the analyzed value.

### Ion Concentrations and pH Value in Blood

At the end of trial, 2 hens from each replicate were randomly selected to measure ion concentrations and pH value in blood. The blood samples were drawn into heparinized tubes from the brachial vein within 2 h after laying. Na^+^, Cl^−^, K^+^ and Ca^2+^ concentrations and pH value in whole blood were tested within 20 minutes after sampling using the automatic blood analyzer (PL2000, Pulang Nanjing Medical Equipment Co., Ltd., Jiangsu, China).

### Renal Function

At the end of trial, the same hens as “*Ion Concentrations and pH Value in Blood*” were selected to measure the contents of creatinine and uric acid in serum, then these hens from the control group and the lowest dietary Cl (0.15%) groups were sacrificed to observe the renal histopathology. The blood samples were drawn into serum separator tubes from the brachial vein within 2 h after laying and placed in the water bath (37°C) for 4 h to harvest serum. The serum was removed from the tube and stored at -20°C until analysis. Fully automatic biochemical analyzer (Zhuoyue 300, Shanghai Kehua Bio-Engineering Co., Ltd., Shanghai, China) was used to measure the contents of creatinine and uric acid in serum. Renal morphology was observed before the tissue was removed. Renal histopathology changes were observed in accordance with the method described by Jadhav et al. (2007). Tissue samples of kidney were first fixed in formalin over 24 h and embedded in paraffin blocks. Then, the blocks cut into 4-µm thickness sections and stained by hematoxylin–eosin (H&E) for histopathological observation using an Olympus BX43 microscope (Olympus Corp., Tokyo, Japan).

### Laying Performance and Eggshell Quality

Egg number and egg weight were recorded daily. Feed consumption was recorded every 4 wk. Average egg weight, ADFI, feed conversion ratio (**FCR**) and mortality rate were calculated at 4-wk intervals during the experimental period. 6 eggs from each replicate were collected daily on the last 3 d in each 4-wk period to measure eggshell quality. Eggshell thickness was measured at the equator and both poles with Egg Shell Thickness Gauge (Ramat Hasharon, Israel Orka Food Technology Ltd., Ramat Hasharon, Israel), and the eggshell thickness was calculated by the average measurements of 3 points. Egg Force Reader (Ramat Hasharon, Israel Orka Food Technology Ltd., Ramat Hasharon, Israel) was used to test eggshell breaking strength. Eggshell weight was weighted after removing egg albumen and the dirt. Eggshell weight ratio was defined by ([eggshell weight]/[egg weight]) × 100%.

### Eggshell Ultrastructure

At the end of trial, 6 eggshells per replicate were collected to image eggshell ultrastructure using scanning electronic microscopy (UHR FE-SEM SU8000, Hitachi Co., Ltd., Tokyo). Two pieces of approximately 0.5 cm * 1 cm eggshell were selected from equatorial section of each eggshell sample and cleaned with distilled water, then dried and glued in the aluminium plate, covered gold power, and imaged by scanning electronic microscopy. Effective layer thickness, mammillary layer thickness and the mammillary knobs width were defined as described by Dunn et al. (2012) and measured under a visual field of 180 × magnification.

### Apparent Ca Metabolizability of Hens, the Ca Content and Total Ca of Eggshell

At the end of the trial, 6 eggshells from each replicate were mixed into a sample to determine the Ca content of the eggshell. The eggshell samples were washed in water and dried at room temperature over 2 d. Each dried sample was weighted as *W_1_* and crushed into powder. Approximately 0.5 g of eggshell powder was put into a burning cup with 3 mL nitric acid and 3 mL H_2_O_2_ and stood for 2 h. Then, sampled cups were digested using a microwave digestion instrument (MDS-10, Shanghai Xinyi Instrument Technology co., Ltd, Shanghai, China). The Ca content in eggshell was analyzed as ***C_1_*** by flame atomic absorption spectrophotometry (Z2000, Hitachi Co., Ltd., Tokyo, Japan). The total Ca of eggshell from each replicate was measured as W_1_* *C_1_*.

Apparent Ca metabolizability in the current study was determined using total fecal collection method according to Yang et al. (2014). 1 cage (3 birds) was selected randomly from each replicate at the end of the trial. Test diets were given for 3 d after the initial 24-h fasting period, and total excreta was collected during the 3 d and stored in sealed bags at -20°C. Remaining feed and feathers in the excreta trays were carefully removed. Excreta collected per cage during the 3-d collection period were pooled and represented 1 replicate, resulting in 6 samples for each treatment. Feed intake and excreta of each replicate were weighed and recorded during the 3-d collection period. Before chemical analysis, excreta samples were thawed and dried at 65°C for 72 h, and finely ground to pass through a 0.5-mm screen. The Ca contents and total Ca excretion were measured using the dried samples as described by the method above (“*Ca Contents and Total Ca of Eggshell*”). The apparent Ca metabolizability of hens was calculated as follow:

$$\text{Apparent}\;\text{Ca}\;\text{metabolizability}\;=\;\left[\left(Ca_{Diet}\right)-\left(Ca_{Excreta}\right)\right]/\left(Ca_{Diet}\right)\times 100\%$$

Where *Ca*_*Diet*_ and *Ca*_*Exreta*_ (g/kg DM) = total Ca contents in the diet and excreta, respectively.

### Statistical Analysis

All analyses were performed using SPSS 25.0 for windows (SPSS Inc., Chicago, IL). The homogeneity of variances was tested at first and the Shapiro Wilk test was used to analyze normality of the data. The data were analyzed using one-way ANOVA and the means were compared using Duncan's multiple range test among the whole groups. To estimate the interaction effects between dietary Cl levels and salt substitutes, data without the control group were analyzed using the GLM procedure of SPSS appropriate for a 2 × 2 factorial arrangement of treatments. Dietary Cl levels (0.21% and 0.15%) and salt substitutes (NaHCO_3_ and Na_2_SO_4_) were fixed factors. For freeing some degrees of freedom and improving the power of this analysis, the interaction effects in the model were removed and main effects of salt substitutes were reanalyzed when the interaction effects were not significant (*P* > 0.05). Finally, the means of dietary Cl levels (0.27%, 0.21% and 0.15%) were analyzed using one-way ANOVA and compared using Duncan's multiple range test. The significant differences were defined as *P* < 0.05. Data are expressed as the mean and pooled SEM.

## RESULTS

### Ions Concentrations and pH Value in Blood

Table 3 demonstrates the effects of dietary Cl reduction by substitution of NaHCO_3_ or Na_2_SO_4_ for part of dietary salt on ions concentrations and pH value in blood of laying hens. No significant differences in Na^+^, Cl^−^, K^+^ and Ca^2+^ concentrations and pH value were observed in the blood (*P* > 0.05).

**Table 3:** Effects of dietary chloride reduction by substitution of sodium bicarbonate (NaHCO_3_) or sodium sulfate (Na_2_SO_4_) for dietary salt on ion concentrations in blood of laying hens (40 wk of age)^1^

Dietary Cl level	Na^+^ (mmol/L)	Cl^−^ (mmol/L)	K^+^ (mmol/L)	Ca^2^^+^ (mmol/L)	pH
0.27% (control)	139.27	106.79	3.98	1.44	7.56
NaHCO_3_					
0.21%	141.29	105.03	4.09	1.40	7.52
0.15%	143.66	106.52	4.11	1.43	7.56
Na_2_SO_4_					
0.21%	138.98	108.70	4.04	1.44	7.56
0.15%	138.98	106.78	4.02	1.43	7.53
SEM^2^	0.86	0.58	0.03	0.02	0.01
ANOVA					
*P*-value	0.36	0.42	0.81	0.98	0.92
Source effect					
NaHCO_3_	142.48	105.78	4.10	1.42	7.54
Na_2_SO_4_	138.98	107.74	4.03	1.43	7.55
SEM^3^	0.86	0.72	0.06	0.02	0.02
Level × source	0.21	0.64	0.86	0.58	0.25
*P*-value	0.55	0.13	0.28	0.39	0.92
Level effect					
0.27%	139.27	106.79	3.98	1.44	7.56
0.21%	140.13	106.87	4.06	1.43	7.54
0.15%	141.32	106.65	4.07	1.43	7.55
SEM^4^	0.64	0.47	0.03	0.01	0.01
*P*-value	0.14	0.86	0.39	0.81	0.84

1 Means of 6 replicates (2 hens per replicate) per treatment.

2 n = 30, df = 29.

3 n = 24, df = 23.

4 n = 30, df = 29.

### Renal Function

**Figure 1:**
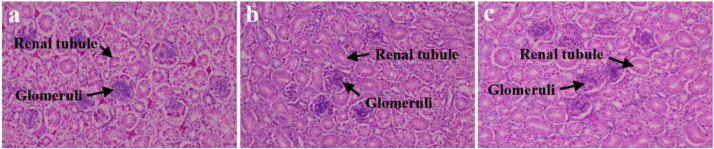
Representative renal histopathological sections in laying hens (40 wk of age; stained by hematoxylin and eosin; magnified 200 ×). (A) Renal histopathological in dietary 0.27% Cl level group. (B, C) Renal histopathological in dietary 0.15% Cl level groups (b, sodium bicarbonate; c, sodium sulfate).

**Figure 2:**
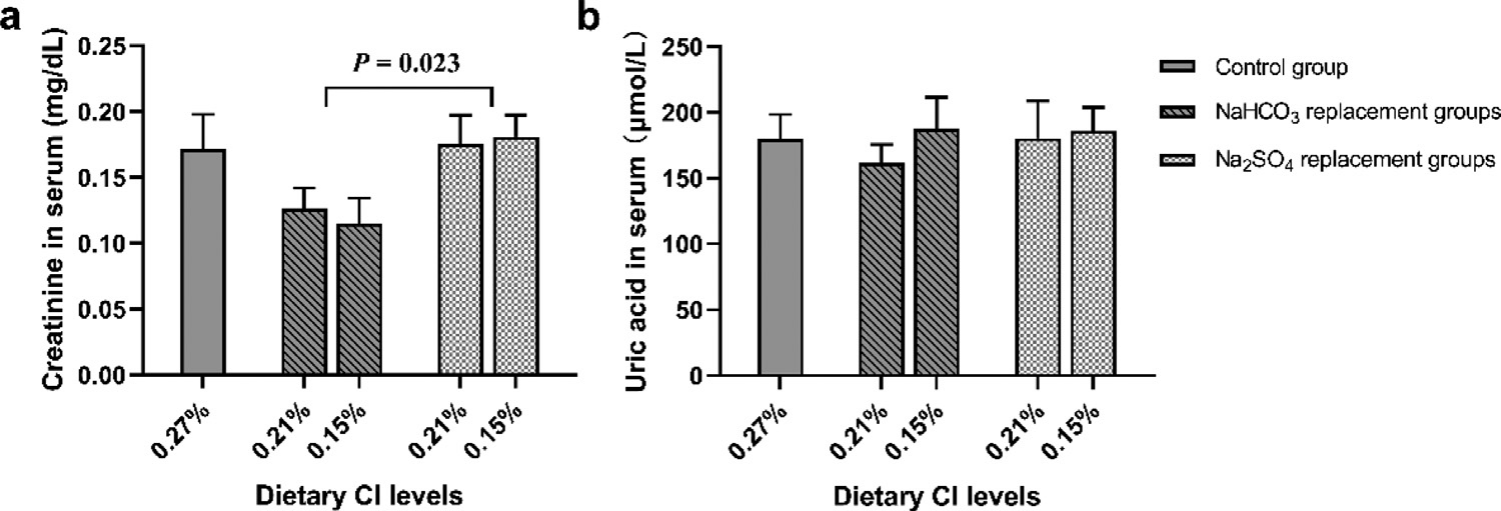
Effects of dietary chloride reduction by substitution of sodium bicarbonate (NaHCO_3_) or sodium sulfate (Na_2_SO_4_) for dietary salt on the contents of creatinine and uric acid in serum of laying hens (40 wk of age; a, creatinine; b, uric acid).

Histopathological sections of kidney are shown in Figure 1. No histopathological changes were observed in the control group (0.27% Cl) and the lowest dietary Cl (0.15%) groups. Dietary Cl reduction did not significantly affect the contents of serum creatinine and uric acid (*P* > 0.05, Figure 2). However, the serum creatinine content of birds in NaHCO_3_ replacement groups was lower than that of birds in Na_2_SO_4_ replacement groups (*P* = 0.023, Figure 2).

### Laying Performance

The effects of dietary Cl reduction by substitution of NaHCO_3_ or Na_2_SO_4_ for part of NaCl on egg production, average egg weight, ADFI and FCR are listed in Table 4 and 5. In comparison with the control group (0.27% Cl), higher egg production was observed in Na_2_SO_4_ replacement groups (*P* = 0.047), and higher ADFI was observed in all replacement groups during wk 37 to 40 of age (*P* < 0.001). Compared with the level of dietary 0.27% Cl, dietary 0.21% and 0.15% Cl levels significantly increased the egg production during wk 33 to 36 (*P* = 0.043), 37 to 40 (*P* = 0.024) and 29 to 40 of age (*P* = 0.023) and raised ADFI during wk 37 to 40 (*P* < 0.001) and 29 to 40 of age (*P* = 0.030). During wk 33 to 36 of age, birds fed the diets containing 0.15% Cl exhibited higher ADFI than those fed dietary 0.27% Cl level (*P* = 0.049). Besides, compared with groups of dietary 0.27% and 0.21% Cl levels, higher FCR occurred in groups of dietary 0.15% Cl level during wk 37 to 40 of age (*P* = 0.028). No significant differences in laying performance were observed between Na_2_SO_4_ and NaHCO_3_ replacement groups (*P* > 0.05).

**Table 4:** Effects of dietary chloride reduction by substitution of sodium bicarbonate (NaHCO_3_) or sodium sulfate (Na_2_SO_4_) for dietary salt on egg production and average egg weight of laying hens (29 to 40 wk of age)^1^

	Egg production (%)				Average egg weight (g)			
Dietary Cl level	29 to 32 wk	33 to 36 wk	37 to 40 wk	29 to 40 wk	29 to 32 wk	33 to 36 wk	37 to 40 wk	29 to 40 wk
0.27% (control)	94.99	91.91	90.79^B^	92.57	60.67	62.92	63.21	62.27
NaHCO_3_								
0.21%	95.54	94.44	92.44^AB^	94.14	60.80	62.78	62.72	62.10
0.15%	96.13	94.10	92.28^AB^	94.07	61.48	63.07	62.32	62.05
Na_2_SO_4_								
0.21%	96.43	95.49	94.91^A^	95.61	60.91	62.75	62.52	62.06
0.15%	94.74	96.43	93.88^A^	95.01	60.80	62.51	62.44	62.27
SEM^2^	0.38	0.52	0.47	0.34	0.17	0.18	0.18	0.14
ANOVA								
*P*-value	0.60	0.065	0.047	0.058	0.62	0.91	0.61	0.98
Source effect								
NaHCO_3_	95.84	94.27	92.36	94.11	61.14	62.93	62.52	62.08
Na_2_SO_4_	95.59	95.96	94.40	95.31	60.86	62.63	62.48	62.17
SEM^3^	0.62	0.80	0.79	0.50	0.25	0.23	0.29	0.22
Level × source	0.21	0.57	0.70	0.67	0.28	0.65	0.71	0.59
*P*-value	0.78	0.15	0.083	0.12	0.43	0.80	0.93	0.51
Level effect								
0.27%	94.99	91.92^b^	90.79^b^	92.57^b^	60.67	62.92	63.21	62.26
0.21%	95.98	94.96^a^	93.67^a^	94.87^a^	60.86	62.76	62.62	62.08
0.15%	95.44	95.26^a^	93.08^a^	94.54^a^	61.14	62.80	62.38	62.10
SEM^4^	0.38	0.52	0.40	0.33	0.17	0.17	0.18	0.14
*P*-value	0.62	0.043	0.024	0.023	0.58	0.95	0.26	0.90

1 Means of 6 replicates (12 hens per replicate) per treatment.

2 n = 30, df = 29.

3 n = 24, df = 23.

4 n = 30, df = 29.

AB Means within a column with no common superscripts differ significantly (*P* < 0.05). ^a,b^Means within a column with no common superscripts differ significantly (*P* < 0.05).

**Table 5:** Effects of dietary chloride reduction by substitution of sodium bicarbonate (NaHCO_3_) or sodium sulfate (Na_2_SO_4_) for dietary salt on ADFI and feed conversion ratio of laying hens (29 to 40 wk of age)^1^

	ADFI (g/hen/d)				FCR^2^ (g/g)			
Dietary Cl level	29 to 32 wk	33 to 36 wk	37 to 40 wk	29 to 40 wk	29 to 32 wk	33 to 36 wk	37 to 40 wk	29 to 40 wk
0.27% (control)	121.94	120.40	114.26^B^	118.87	2.10	2.07	1.97	2.05
NaHCO_3_								
0.21%	121.30	121.94	116.91^A^	120.05	2.07	2.03	1.97	2.02
0.15%	121.23	121.70	116.99^A^	119.82	2.05	2.05	2.04	2.05
Na_2_SO_4_								
0.21%	120.24	120.88	117.05^A^	119.39	2.04	2.00	1.95	2.00
0.15%	121.57	121.86	116.29^A^	120.30	2.13	2.07	2.02	2.08
SEM^3^	0.23	0.25	0.26	0.17	0.01	0.02	0.01	0.01
ANOVA								
*P*-value	0.21	0.079	<0.001	0.11	0.14	0.73	0.12	0.16
Source effect								
NaHCO_3_	121.27	121.82	116.95	119.94	2.06	2.04	2.01	2.03
Na_2_SO_4_	120.91	121.37	116.67	119.85	2.09	2.04	1.99	2.04
SEM^4^	0.37	0.39	0.28	0.27	0.02	0.03	0.02	0.02
Level × source	0.19	0.28	0.31	0.44	0.17	0.58	0.97	0.33
*P*-value	0.50	0.42	0.50	0.35	0.40	0.94	0.57	0.95
Level effect								
0.27%	121.94	120.39^b^	114.26^b^	118.87^b^	2.11	2.06	1.97^b^	2.05
0.21%	120.77	121.41^ab^	116.98^a^	119.72^a^	2.05	2.01	1.96^b^	2.01
0.15%	121.40	121.78^a^	116.64^a^	119.94^a^	2.09	2.06	2.03^a^	2.06
SEM^5^	0.23	0.21	0.26	0.15	0.01	0.02	0.01	0.01
*P*-value	0.17	0.049	<0.001	0.030	0.28	0.41	0.028	0.10

1 Means of 6 replicates (12 hens per replicate) per treatment.

2 FCR, feed conversion ratio (feed/egg, g/g).

3 n = 30, df = 29.

4 n = 24, df = 23.

5 n = 30, df = 29. ^A-B^Means within a column with no common superscripts differ significantly (*P* < 0.05).

ab Means within a column with no common superscripts differ significantly (*P* < 0.05).

### Eggshell Quality

As shown in Table 6, in both NaHCO_3_ and Na_2_SO_4_ replacement groups, eggshell breaking strength of birds fed the diets containing 0.15% Cl (*P* = 0.029) and thickness of birds fed the diets containing 0.21% Cl (*P* = 0.021) were significantly higher than those of the control group (0.27% Cl) at the end of wk 40 of age. At the end of wk 36 of age, the eggshell breaking strength of laying hens in dietary 0.15% Cl group was significantly increased compared with that of laying hens fed with dietary 0.27% Cl (*P* = 0.031). With the decrease of dietary Cl, eggshell breaking strength (*P* = 0.011) and eggshell thickness (*P* = 0.011) were significantly increased at the end of wk 40 of age. During the whole trial, no significant differences were detected in eggshell weight among all treatments (Table 7, *P* > 0.05). However, as shown in Table 7, the eggshell weight ratio was significantly increased with dietary Cl reduction at wk 36 (*P* = 0.034) and 40 (*P* = 0.049) of age. There were no significant differences in eggshell quality between Na_2_SO_4_ and NaHCO_3_ replacement groups (Table 6 & 7, *P* > 0.05).

**Table 6:** Effects of dietary chloride reduction in laying hen diets by substitution of sodium bicarbonate (NaHCO_3_) or sodium sulfate (Na_2_SO_4_) for dietary salt on eggshell breaking strength and thickness (28 to 40 wk of age)^1^

	Breaking strength (N)				Thickness (mm)			
Dietary Cl level	28 wk	32 wk	36 wk	40 wk	28 wk	32 wk	36 wk	40 wk
0.27% (control)	46.77	46.99	46.41	43.53^B^	0.45	0.44	0.43	0.44^B^
NaHCO_3_								
0.21%	45.34	45.24	47.27	46.14^A^	0.44	0.43	0.43	0.46^A^
0.15%	45.90	45.66	48.60	45.63^A^	0.44	0.44	0.43	0.45^AB^
Na_2_SO_4_								
0.21%	45.93	46.32	47.74	45.23^AB^	0.44	0.44	0.43	0.46^A^
0.15%	46.33	46.14	50.00	46.62^A^	0.45	0.44	0.44	0.47^A^
SEM^2^	0.39	0.26	0.48	0.34	0.0015	0.0015	0.0013	0.0032
ANOVA								
*P*-value	0.84	0.29	0.093	0.029	0.48	0.41	0.075	0.021
Source effect								
NaHCO_3_	45.62	45.45	47.94	45.89	0.44	0.44	0.43	0.46
Na_2_SO_4_	46.13	46.23	48.87	45.93	0.45	0.44	0.44	0.47
SEM^3^	0.52	0.42	0.64	0.46	0.0018	0.0021	0.0019	0.0021
Level × source	0.51	0.61	0.61	0.16	0.81	0.22	0.83	0.14
*P*-value	0.15	0.21	0.31	0.96	0.29	0.59	0.78	0.87
Level effect								
0.27%	46.77	46.99	46.41^b^	43.53^b^	0.45	0.44	0.43	0.43^b^
0.21%	45.64	45.78	47.51^ab^	45.69^a^	0.44	0.44	0.43	0.43^b^
0.15%	46.12	45.90	49.30^a^	46.13^a^	0.44	0.44	0.43	0.44^a^
SEM^4^	0.35	0.26	0.44	0.34	0.0012	0.0013	0.0013	0.0014
*P*-value	0.44	0.22	0.031	0.011	0.54	0.26	0.14	0.011

1 Means of 6 replicates (18 eggs per replicate) per treatment.

2 n = 30, df = 29.

3 n = 24, df = 23.

4 n = 30, df = 29.

AB Means within a column with no common superscripts differ significantly (*P* < 0.05).

ab Means within a column with no common superscripts differ significantly (*P* < 0.05).

**Table 7:** Effects of dietary chloride reduction in laying hen diets by substitution of sodium bicarbonate (NaHCO_3_) or sodium sulfate (Na_2_SO_4_) for dietary salt on eggshell weight and ratio (28 to 40 wk of age)^1^

	Eggshell weight (g)				Eggshell Ratio (%)			
Dietary Cl level	28 wk	32 wk	36 wk	40 wk	28 wk	32 wk	36 wk	40 wk
0.27% (control)	6.23	6.08	6.15	6.09	10.14	9.77	9.58	9.73
NaHCO_3_								
0.21%	6.27	5.91	6.17	6.17	10.21	9.64	9.51	9.83
0.15%	6.19	6.06	6.22	6.29	10.10	9.66	9.75	9.98
Na_2_SO_4_								
0.21%	6.14	6.06	6.04	6.13	9.98	9.71	9.51	9.89
0.15%	6.26	6.11	6.21	6.23	10.27	9.83	9.75	10.06
SEM^2^	0.24	0.18	0.14	0.19	0.31	0.18	0.23	0.26
ANOVA								
*P*-value	0.88	0.36	0.19	0.44	0.47	0.31	0.16	0.18
Source effect								
NaHCO_3_	6.23	5.99	6.20	6.23	10.16	9.65	9.63	9.91
Na_2_SO_4_	6.20	6.09	6.13	6.18	10.13	9.77	9.63	9.98
SEM^3^	0.06	0.05	0.04	0.05	0.07	0.06	0.07	0.07
Level × source	0.25	0.45	0.27	0.95	0.072	0.57	0.96	0.93
*P*-value	0.74	0.13	0.21	0.51	0.80	0.15	0.99	0.52
Level effect								
0.27%	6.23	6.08	6.15	6.09	10.14	9.77	9.58^ab^	9.72^b^
0.21%	6.21	5.99	6.11	6.15	10.09	9.68	9.51^b^	9.86^ab^
0.15%	6.23	6.09	6.21	6.26	10.19	9.75	9.75^a^	10.02^a^
SEM^4^	0.04	0.03	0.02	0.03	0.05	0.04	0.04	0.05
*P*-value	0.98	0.35	0.17	0.15	0.72	0.57	0.034	0.049

1 Means of 6 replicates (18 eggs per replicate) per treatment.

2 n = 30, df = 29.

3 n = 24, df = 23.

4 n = 30, df = 29.

ab Means within a column with no common superscripts differ significantly (*P* < 0.05).

### Eggshell Ultrastructure

**Figure 3:**
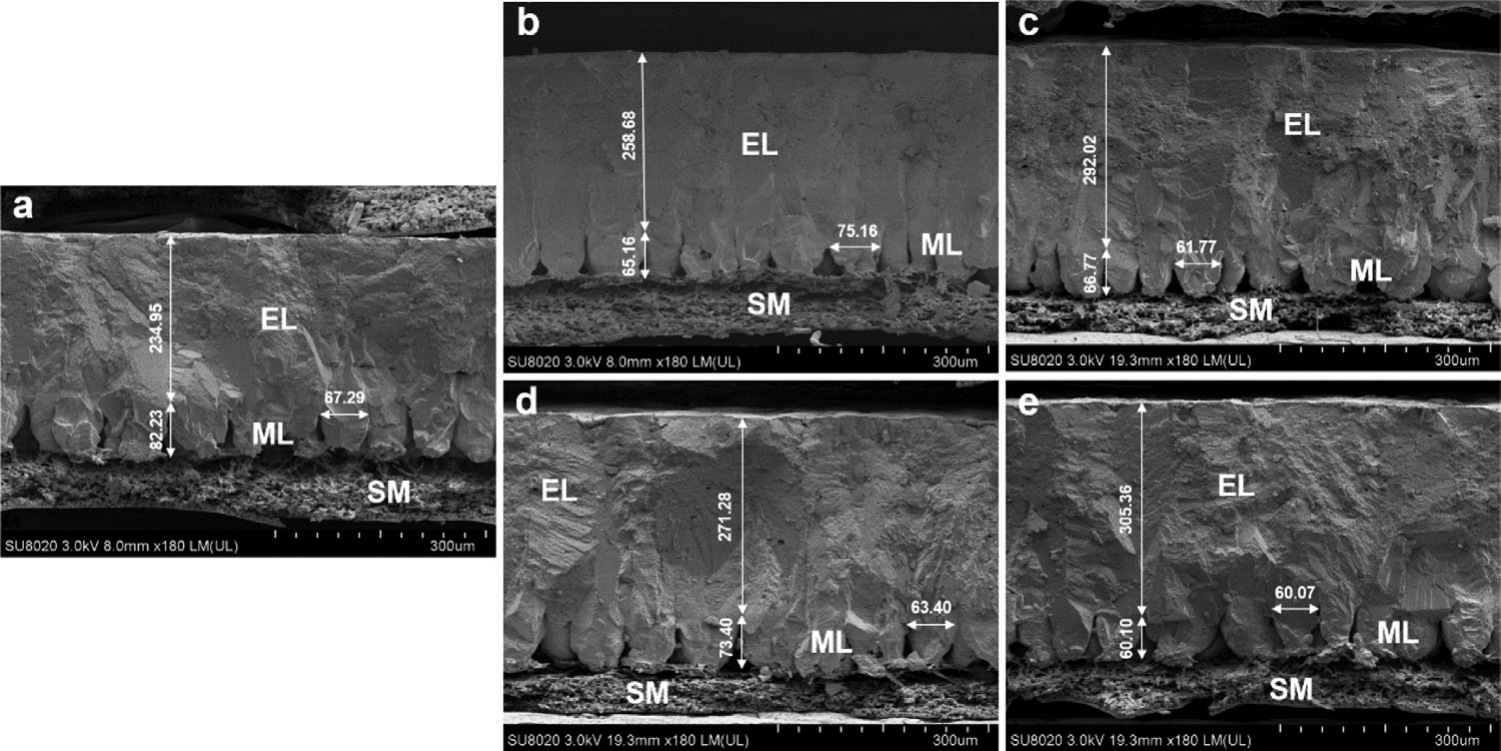
Scanning electron microscope images of vertical profiles of eggshells. (A) The vertical ultrastructure of eggshell in dietary 0.27% Cl level group. (B, C) The vertical ultrastructure of eggshell with dietary Cl level at 0.21% and 0.15% in sodium bicarbonate replacement groups, respectively. (D, E) The vertical ultrastructure of eggshell with dietary Cl level at 0.21% and 0.15% in sodium sulfate replacement groups, respectively. Abbreviations: EL, effective layer; ML, mammillary layer; SM, shell membrane.

Scanning electron microscopy images in Figure 3 shows the eggshell ultrastructure of laying hens fed diets containing 0.15%, 0.21% and 0.27% Cl. Compared with that of birds in dietary 0.27% Cl group, the mammillary layer thickness of birds fed with dietary 0.21% and 0.15% Cl was significantly decreased (Table 8, *P* = 0.002), and the effective layer thickness of birds fed diets containing 0.15% Cl was significantly increased (Table 8, *P* = 0.001) in both NaHCO_3_ and Na_2_SO_4_ replacement groups. Eggshell effective layer thickness was significantly increased (Table 8, *P* = 0.001), and mammillary layer thickness was significantly decreased with dietary Cl reduction (Table 8, *P* < 0.001). Besides, mammillary knob width was not significantly affected by the dietary Cl levels (Table 8, *P* > 0.05), but Na_2_SO_4_ replacement groups had wider mammillary knob width compared with NaHCO_3_ replacement groups (Table 8, *P* = 0.038).

**Table 8:** Effects of dietary chloride reduction in laying hen diets by substitution of sodium bicarbonate (NaHCO_3_) or sodium sulfate (Na_2_SO_4_) for dietary salt on eggshell ultrastructure (40 wk of age)^1^^.^

Dietary Cl level	Effective layer thickness (μm)	Mammillary layer thickness (μm)	Mammillary knob width (μm)
0.27% (control)	260.64^C^	64.55^A^	74.00
NaHCO_3_			
0.21%	274.10^BC^	57.09^B^	69.89
0.15%	288.81^AB^	51.47^B^	73.32
Na_2_SO_4_			
0.21%	274.57^BC^	56.43^B^	74.47
0.15%	294.70^A^	54.31^B^	74.80
SEM^2^	3.09	1.17	0.76
ANOVA			
*P*-value	0.001	0.002	0.25
Source effect			
NaHCO_3_	281.46	54.28	71.60^b^
Na_2_SO_4_	284.64	55.37	74.64^a^
SEM^3^	3.77	1.48	0.96
Level × source	0.62	0.41	0.27
*P*-value	0.56	0.61	0.038
Level effect			
0.27%	260.64^c^	64.55^a^	74.00
0.21%	274.34^b^	56.76^b^	72.18
0.15%	291.76^a^	52.89^b^	74.06
SEM^4^	2.89	1.17	0.86
*P*-value	0.001	<0.001	0.24

1 Means of 6 replicates (6 eggs per replicate) per treatment.

2 n = 30, df = 29.

3 n = 24, df = 23.

4 n = 30, df = 29. ^A-C^Means within a column with no common superscripts differ significantly (*P* < 0.05). ^a-c^ Means within a column with no common superscripts differ significantly (*P* < 0.05).

### Apparent Ca Metabolizability of Hens, the Ca Content and Total Ca of Eggshell

As shown in Table 9, apparent Ca metabolizability of hens in dietary 0.15% Cl groups (in both NaHCO_3_ and Na_2_SO_4_ replacement groups) was significantly enhanced compared with that of hens in dietary 0.27% Cl group (*P* = 0.008). With the decrease of dietary Cl levels, apparent Ca metabolizability of hens (*P* = 0.002) and total Ca of eggshell (*P* = 0.046) were significantly increased. However, dietary Cl levels did not significantly affect the Ca content of shell (*P* > 0.05). No significant differences in apparent Ca metabolizability of hens, the Ca content and total Ca of eggshell were observed between Na_2_SO_4_ and NaHCO_3_ replacement groups (*P* > 0.05).

**Table 9:** Effects of dietary chloride reduction by substitution of sodium bicarbonate (NaHCO_3_) or sodium sulfate (Na_2_SO_4_) for dietary salt on Ca metabolism of laying hens (40 wk of age)^1^

Dietary Cl level	Apparent metabolizability of Ca (%)	The Ca contents of shell (%)	Total Ca of shell (g)
0.27% (control)	40.07^C^	34.14	2.08
NaHCO_3_			
0.21%	44.23^BC^	34.66	2.14
0.15%	48.10^AB^	35.32	2.22
Na_2_SO_4_			
0.21%	45.94^ABC^	35.46	2.17
0.15%	51.92^A^	35.35	2.20
SEM^2^	1.14	0.34	0.02
ANOVA			
*P*-value	0.008	0.71	0.17
Source effect			
NaHCO_3_	46.17	34.99	2.18
Na_2_SO_4_	48.93	35.41	2.19
SEM^3^	1.50	0.50	0.03
Level × source	0.62	0.59	0.54
*P*-value	0.21	0.56	0.86
Level effect			
0.27%	40.07^b^	34.14	2.08^b^
0.21%	45.09^a^	35.06	2.15^ab^
0.15%	50.01^a^	35.33	2.21^a^
SEM^4^	1.14	0.34	0.02
*P*-value	0.002	0.44	0.046

1 Means of 6 replicates (3 hens or 6 eggs per replicate) per treatment.

2 n = 30, df = 29.

3 n = 24, df = 23.

4 n = 30, df = 29. ^AC^Means within a column with no common superscripts differ significantly (*P* < 0.05). ^ac^Means within a column with no common superscripts differ significantly (*P* < 0.05).

## DISCUSSION

The impact on the health of the laying hens should be taken into consideration when part of dietary NaCl was replaced by NaHCO_3_ or Na_2_SO_4_ since their inappropriate addition may cause blood ion disorder and other ionic associated diseases (Davison and Wideman, 1992; Wang et al., 2020). There were no significant changes in the pH value and ion concentrations in blood of layers following dietary Cl reduction at the end of this trial. This may be because that the dietary electrolyte balance values ranged from 164 to 198 mEq/kg when dietary Cl levels changed from 0.27% to 0.15% in the current study, which were within the acceptable range of dietary electrolyte balance value for poultry (150 - 250 mEq/kg) (Ahmad et al., 2009; Mushtaq and Pasha, 2013). Besides, as a main organ for ion reabsorption, the kidneys of layers presented no obvious injures according to the observation of pathological section, which was consistent with no significant changes in serum creatinine and uric acid contents. Thus, in the current study, the health status of layers was not impaired when part of NaCl was replaced by NaHCO_3_ or Na_2_SO_4_ to reduce dietary Cl levels.

The substitution of NaHCO_3_ or Na_2_SO_4_ for partial NaCl in laying hen diets was not only harmless to the health of layers, but also beneficial to laying performance. It was evidenced by increased egg production and feed intake in both NaHCO_3_ and Na_2_SO_4_ replacement groups when the dietary Cl levels were decreased from 0.27% to 0.21% and 0.15%. However, similar results were not found in another study (Wang et al., 2020), in which the substitution of partial dietary NaCl by Na_2_SO_4_ could not affect performance. A possible reason for this difference may be that the hens we tested were in the peak laying period and younger. Jiang et al. (2015) reported that dietary Cl reduction, by substituting NaHCO_3_ for part of NaCl, could improve laying rate of young hens (wk 25 - 45 of age and wk 25 - 50 of age), but was not beneficial for hens after wk 50 of age. Therefore, the effect of dietary Cl reduction on egg production may be related to the age of laying hens, and improvement effects tended to occur in young layers. Analogously, an increased ADFI was also observed in birds receiving diets with low Cl levels in both NaHCO_3_ and Na_2_SO_4_ replacement groups. There was evidence that increased laying rate would be expected to a coinstantaneous increase in feed intake (Neijat et al., 2011; Nasr et al., 2013). A significant correlation was observed between the laying rate and ADFI in the current study (*P* = 0.005, R = 0.52). This may indicate that an increase in egg production led to a greater demand for nutrients and consequently inducing an increased feed intake. Based on these results, an increase in FCR was observed in dietary 0.15% Cl groups during wk 37 to 40, which can be partially explained by increased ADFI or can be related to a tendency of lower egg weight (not significant).

Broken and soft-shelled eggs are detrimental to the economic benefits in the layer industry and are considered to be related to poor eggshell breaking strength (Dunn, 2011). Herein, we found eggshell breaking strength was enhanced when NaCl was partially replaced by NaHCO_3_ or Na_2_SO_4_ to reduce dietary Cl levels. Eggshell breaking strength depends not only on its thickness, weight and weight ratio but also on its ultrastructure (Roberts and Brackpool, 1994). Similar to previous studies (Faria et al., 2000; Wang et al., 2020; Jiang et al., 2015 ), increased eggshell thickness was observed in dietary 0.15% Cl groups in the current study, implying a possible enhancement in calcification process of eggshell following dietary Cl reduction. Additionally, the improvements of eggshell breaking strength and thickness may be related to the better eggshell ultrastructure in lower dietary Cl groups, characterized by thicker effective layer thickness and smaller mammillary layer thickness. The effective layer, including palisade layer, vertical crystal layer and cuticle (Fathi et al., 2007), is the main mineralized structure of eggshell and dominates the eggshell mechanical characteristics (Radwan, 2016). The thicker effective layer is vital for the eggshell to resist the inception and propagation of cracks (Zhang et al., 2017). Improvements of effective layer thickness can be attributed to earlier fusion of the mammillae and adequate supply of calcium carbonate (**CaCO_3_**) during the formation of the effective layer. It can be confirmed by the decreased thickness of mammillary layer (Dunn et al., 2012) and the greater increase in the thickness of the effective layer than the decrease of the mammillary layer in the current study. Besides, the higher increase in effective layer thickness may be a reason of the increased eggshell thickness in groups fed with lower dietary Cl levels. Mammillary knob width is an indicator to evaluate mammillae density. An ordered and compact mammillary layer is more conducive to resisting external force (Bain, 1992) via providing a firm foundation for the formation of the effective layer (Carnarius et al., 1996). Therefore, the effective layer of eggshell is still the most direct impact portion of the shell strength (Carnarius et al., 1996). Although the NaHCO_3_ replacement groups exhibited a narrower mammillary knob width than Na_2_SO_4_ replacement groups, there were no significant differences in effective layer thickness between the two substitution strategies. This might explain why the two substitutes affected mammillary knob width but not the eggshell mechanical characteristics in the current study.

During eggshell formation, Ca deposition begins around sites of mammillae nucleation on the outer shell membrane (Stemberger et al., 1977). The CaCO_3_ accumulates around these nucleation sites, forming the calcified structure of eggshell, including mammillary layer and effective layer (Creger et al., 1976). Based on the results of mechanical characteristics and ultrastructure of eggshell, we found better calcification structures were presented in the low Cl groups, presenting as increased effective layer thickness and decreased mammillary layer thickness as well as increased total eggshell thickness. The CaCO_3_ is the main component of the calcified structure in eggshells (Nys and Guyot, 2011). The Ca content and the total Ca of shells were measured and calculated in order to investigate the improvements of eggshell calcification in dietary low Cl groups. Although we failed to observe significant differences in the Ca content of eggshell and eggshell weight, the total Ca of eggshell was increased with dietary Cl reduction, evidencing the more adequate supply of CaCO_3_ was exerted during the eggshell formation. This also indicated that more Ca^2+^ and HCO_3_^−^ were involved in the eggshell calcification. Consistently, we found that apparent metabolizability of Ca was increased with dietary Cl reduction in both NaHCO_3_ and Na_2_SO_4_ replacement groups. It suggested that decreasing dietary Cl levels by substitution of NaHCO_3_ or Na_2_SO_4_ for part of NaCl could reinforce the absorption capacity of dietary Ca in laying hens, which may be related to the improvements of the intestinal morphology and nutrient absorption by these two substitutes (Jiang et al., 2015; Xiong et al., 2015). Therefore, when NaCl was partially replaced by NaHCO_3_ or Na_2_SO_4_ to reduce dietary Cl levels, the improvements of eggshell calcification could be attributed to the adequate supply of eggshell Ca^2+^ caused by the rises of feed intake and Ca^2+^ absorption during the eggshell formation.

Dietary NaHCO_3_ supplementation could provide HCO_3_^−^ ions necessary for eggshell formation (Jonchère et al., 2012). However, increased HCO_3_^−^ ions did not appear to originate primarily from feed intake during eggshell formation due to similar improvements in the Na_2_SO_4_ replacement groups. It suggested that reducing dietary Cl levels by replacing part of NaCl with NaHCO_3_ or Na_2_SO_4_ could stimulate the autogenous synthesis of HCO_3_^−^ in layers. An alkaline condition is considered more favorable for the generation of HCO_3_^−^ ions (Supuran, 2008). During calcification process of eggshell, Cl^−^ could be output by chloride voltage-gated channel 5 in the basal membrane of glandular cells in exchange for H^+^ entry (Duran et al., 2010), resulting in depressions of acid-base balance related measurements (Hamilton and Thompson, 1980; Junqueira et al., 1984). Hence, in the groups with lower dietary Cl level, increased HCO_3_^−^ was mainly produced by the hydration of carbon dioxide in glandular cells, which was attributed to the provision of a suitable alkaline condition induced by dietary Cl reduction.

Interestingly, compared with NaHCO_3_-fed groups, a tendency for increased laying production was observed in Na_2_SO_4_-fed groups during 37 to 40 wk of age (*P* = 0.083). This may be because less methionine can be spared by cystine in the presence of inorganic sulfate than in its absence, thereby increasing retention of protein and energy (Sasse and Baker, 1974). However, this benefit was related to the content of sulfur-containing amino acids in the diet (Sasse and Baker, 1974). More detailed improvement effects and mechanisms need to be further explored in the future. Apart from this, no significant differences between NaHCO_3_ and Na_2_SO_4_ replacement groups were observed in the improvements of laying performance and eggshell mechanical properties. Quite different from that, our previous study reported that the eggshell quality of layers supplemented with dietary Na_2_SO_4_ was better than that of layers supplemented with dietary NaHCO_3_ when supplementing the same Na level (0.08% - 0.33%) (Fu et al., 2021). The possible reason was that the amounts of NaHCO_3_ and Na_2_SO_4_ supplementation (converted into 0 - 0.08% Na level) in this study were too small to cause significant differences in eggshell quality. Thus, either of NaHCO_3_ and Na_2_SO_4_ can be selected to replace part of NaCl as a suitable strategy of salt addition in laying hen diets, since no significant differences in laying performance and eggshell quality occurred between these two strategies of salt addition.

In conclusion, reducing dietary Cl level from 0.27% to 0.15% by substitution of NaHCO_3_ or Na_2_SO_4_ for part of NaCl could improve laying performance and eggshell quality, and there were no differences in improvements between NaHCO_3_ and Na_2_SO_4_ replacement groups. The improved eggshell quality may be related to increased shell Ca^2+^ and HCO_3_^−^ supply as well as improved eggshell ultrastructure.

## ACKNOWLEDGMENTS

This study was supported by Shandong Key Science and Technology Innovation Program (2019JZZY010704), China Agriculture Research System (CARS-40-K12), Beijing Innovation Consortium of Agriculture Research System (BAIC04-2020) and Agricultural Science and Technology Innovation Program (ASTIP) of the Chinese Academy of Agricultural Sciences.

## DISCLOSURES

The authors declare no conflicts of interest.
